# Asking informed consent may lead to significant participation bias and suboptimal cardiovascular risk management in learning healthcare systems

**DOI:** 10.1186/s12874-023-01924-6

**Published:** 2023-04-22

**Authors:** Anna G. M. Zondag, T. Katrien J. Groenhof, Rieke van der Graaf, Wouter W. van Solinge, Michiel L. Bots, Saskia Haitjema, UCC-CVRM study group, Pim A. de Jong, Marianne C. Verhaar, Frank L. J. Visseren, Folkert W. Asselbergs, Niels P. van der Kaaij, Imo E. Hoefer, Gert J. de Borst, Ynte M. Ruigrok, Monika Hollander, A. Titia Lely, Mariëlle H. Emmelot-Vonk

**Affiliations:** 1 Central Diagnostic Laboratory, Division of Laboratories, Pharmacy and Biomedical Genetics, University Medical Center Utrecht, Utrecht University, Utrecht, the Netherlands; 2 Department of Obstetrics and Gynaecology, St. Antonius Hospital, Utrecht, the Netherlands; 3 Julius Center for Health Sciences and Primary Care, University Medical Center Utrecht, Utrecht University, Utrecht, The Netherlands

**Keywords:** Learning healthcare system, Cardiovascular diseases, Informed consent, Participation bias, CVRM

## Abstract

**Background:**

The Utrecht Cardiovascular Cohort – CardioVascular Risk Management (UCC-CVRM) was set up as a learning healthcare system (LHS), aiming at guideline based cardiovascular risk factor measurement in all patients in routine clinical care. However, not all patients provided informed consent, which may lead to participation bias. We aimed to study participation bias in a LHS by assessing differences in and completeness of cardiovascular risk management (CVRM) indicators in electronic health records (EHRs) of consenting, non-consenting, and non-responding patients, using the UCC-CVRM as an example.

**Methods:**

All patients visiting the University Medical Center Utrecht for first time evaluation of a(n) (a)symptomatic vascular disease or condition were invited to participate. Routine care data was collected in the EHR and an informed consent was asked. Differences in patient characteristics were compared between consent groups. We performed multivariable logistic regression to identify determinants of non-consent. We used multinomial regression for an exploratory analysis for the determinants of non-response. Presence of CVRM indicators were compared between consent groups. A waiver (19/641) was obtained from our ethics committee.

**Results:**

Out of 5730 patients invited, 2378 were consenting, 1907 non-consenting, and 1445 non-responding. Non-consent was related to young and old age, lower education level, lower BMI, physical activity and haemoglobin levels, higher heartrate, cardiovascular disease history and absence of proteinuria. Non-response increased with young and old age, higher education level, physical activity, HbA1c and decreased with lower levels of haemoglobin, BMI, and systolic blood pressure. Presence of CVRM indicators was 5–30% lower in non-consenting patients and even lower in non-responding patients, compared to consenting patients. Non-consent and non-response varied across specialisms.

**Conclusions:**

A traditional informed consent procedure in a LHS may lead to participation bias and potentially to suboptimal CVRM, which is detrimental for feedback on findings in a LHS. This underlines the importance of reassessing the informed consent procedure in a LHS.

**Supplementary Information:**

The online version contains supplementary material available at 10.1186/s12874-023-01924-6.

## Introduction

As cardiovascular diseases are the leading cause of death globally, international cardiovascular risk management (CVRM) guidelines have been developed to reduce cardiovascular morbidity and mortality [1–4]. These guidelines are meant to assist health professionals in the decision making process and include cardiovascular risk indicators that should be assessed in all at-risk patients, such as, smoking status, blood pressure, serum lipids, as well as recommendations for treatment of these risk factors. However, compliance to assessment of cardiovascular risk indicators according to these guidelines varies considerably between treating specialists [1, 5]. Therefore, the Center for Circulatory Health of the University Medical Center (UMC) Utrecht initiated the cross-specialty Utrecht Cardiovascular Cohort-CardioVascular Risk Management (UCC-CVRM) project in 2015. UCC-CVRM aims for hospital-wide uniform assessment and registration of guideline based cardiovascular risk indicators in all patients referred for cardiovascular evaluation in a learning healthcare system (LHS) approach [6]. In addition, broad informed consent was asked for scientific purposes: reuse of healthcare data, including linkage, for scientific research and blood sample storage in a biobank [6, 7].

A LHS is a cycle in which “evidence informs practice, and practice informs evidence” [8]. In other words; routine clinical care data collected from the EHR is analysed and the knowledge generated from these analyses is used to change healthcare practices by, for example, closing the feedback loop to healthcare professionals [8]. An important characteristic of a LHS is its inclusivity, all patients visiting a participating department of the LHS participate [9]. However, when a traditional informed consent procedure is in place, which was the case in UCC-CVRM, only data from consenting patients is processed and used to create new evidence and improve the quality of care. We previously showed that not all UCC-CVRM patients provided informed consent, 57%, potentially impairing the possible benefits of the approach in terms of complete registration and assessment of cardiovascular risk status in all patients [9].

Non-consent is an old but persisting phenomenon with causes ranging from forgetting to return the consent form (more often categorised as “non-response”) to actively stating not wanting to participate at all (more often categorised as “non-consent”) [10]. The clinical significance and consequence of non-consent and non-response may be a misrepresentation of the actual population by only those who provided informed consent, called participation bias [11–13]. Under Dutch law, non-consenting and non-responding patients belong to the same group ‘patients that were asked, yet did not provide written informed consent’. To date, however, because of missing informed consents, information on the characteristics of non-consenting and non-responding individuals within clinical research is limited and, when available, mostly limited to examination of age and sex distributions [12]. Yet, participation bias may lead to under- or overestimation of associations and hampers generalisations and conclusions [14].

Therefore, the aims of this study were to gain insight into the differences between consenting, non-consenting and non-responding patients and the determinants thereof. Furthermore, we aimed to assess the differences in structured registration of cardiovascular risk indicators between the consent groups.

## Methods

We followed the Strengthening the Reporting of Observational Studies in Epidemiology (STROBE) guidelines for observational (cross-sectional) studies. The STROBE statement can be found in Supplement 1 Table 1 (S1T1).

### Study setting

The rationale and design of the ongoing prospective UCC-CVRM cohort study have been described elsewhere [6]. In short, all patients visiting one of the Center for Circulatory Health’s departments at the UMC Utrecht for the first time to evaluate a(n) (a)symptomatic vascular disease or condition were invited to participate. All eligible patients received the invitation via regular mail, including information and the UCC-CVRM questionnaire. Patients filled out a questionnaire on demographics, intoxications, medical history, medication use, physical activity, family cardiovascular disease (CVD) history and pregnancy history before their planned visit, or afterwards, in case of emergency visits. The full questionnaire was added as supplemental material (S2). Additionally, a set of data, based on the recommendations in the Dutch CVRM guidelines, was collected in the EHR as part of routine clinical care. Prior to the appointment with the medical specialist, patients were seen by a trained research nurse to answer any remaining questions regarding the questionnaire. Then, patients were asked if they would consider participation in the UCC-CVRM. Written informed consent was requested for the use of routine clinical care data for scientific research, blood sample storage in the biobank, and linkage with registries for follow-up [6]. All information, including routine care data, questionnaire data and informed consent data were made available through the UMC Utrecht Research Data Platform. All participating departments in the LHS received monthly progress reports about the number of invited patients in the previous month and the percentage of patients that provided written informed consent. In addition, of the patients with a written informed consent, the percentage with a UCC-CVRM questionnaire registered in the EHR and registered laboratory measurements was shown, and the percentage for which we were able to calculate the 10-years risk of CVD morbidity or mortality. The present dataset was based on information collected from May 2015 to December 2019.

### Study population

We restricted the analyses to patients aged 18 years or older with planned visits at an outpatient clinic. We excluded specialties with less than 10 patients in either the consent, non-consent, or non-response group. This was the case for the vascular surgery, cardiothoracic surgery, infectious diseases, obstetrics and the multidisciplinary cardiovascular department. Besides, the obstetrics and gynaecology specialisms were excluded because we wanted to study sex-differences and including these departments would lead to an overrepresentation of women. The neurology department did not have an outpatient clinic and was, therefore, excluded. Additionally, only consenting or non-consenting patients up until December 31^st^ 2019 were included in the analyses because of complete change in health care during the COVID-19 pandemic in 2020 [15]. The non-response group consisted of patients that were invited to participate in the UCC-CVRM, but never responded to the invitation. For them the invitation date was used to select the patients up until December 31^st^, 2019.

### Variable source and definition

Sex, blood pressure, BMI, heart rate and laboratory measurements were extracted from structured fields in the EHR. For blood pressure the measurement ± 7 days of the UCC-CVRM inclusion date was used, for all other measurements the value closest to inclusion date ± 21 days. Measurements outside of these cut-offs were not considered clinically relevant for the patient’s visit to the UMC Utrecht. In these cases, a missing value was recorded. Age was calculated at inclusion. Self-reported data from the UCC-CVRM questionnaire was used to obtain information on education level, smoking status, cardiovascular history, and other cardiovascular risk factors. Hyperlipidemia was defined as having an LDL-cholesterol level higher than 3 mmol/L. We defined ‘high education’ as having obtained a degree from a University of Applied Sciences or general University. Furthermore, patients were asked if they were diagnosed with diabetes, excluding gestational diabetes. Total activity (METminutes) per week was calculated using self-reported data from the validated SQUASH questionnaire [16]. Absolute 10-year risk scores for cardiovascular disease (CVD) morbidity and mortality were calculated for each patient using prevailing algorithms at the time of the start of UCC-CVRM, being either the SCORE risk model (SCORE-NL) [17], United Kingdom Prospective Diabetes Study risk score (UKPDS) [18], or the Second Manifestations of Arterial Disease (SMART) score [19], depending on the patient’s medical history.

### Data analyses

We presented the patient characteristics in strata of consent, non-consent, and non-response in number and percentages, as means and standard deviations or medians and the first and third quartiles, as appropriate. Additionally, we presented the sex distribution within age categories and treating specialties, stratified by consent status.

Due to the nature and large amount of missing data in the non-response group, we decided to exclude them from the regression analyses exploring determinants of non-consent, and thus proceeded with presentation of two groups only (see S3T1). Potential determinants of non-consent were selected based on literature and findings of descriptive statistics. For the analyses regarding the department, we took cardiology as a reference since most patients were recruited from that department. For age, we took the 70–79 year group as a reference since that was the age group with the largest number of invited subjects. Multiple imputation was used to deal with missing values before fitting the multivariable logistic regression model, using the MICE package in R [20]. To avoid biased imputations, variables were imputed separately by consent status. We used backward selection to fit the logistic regression model and pooled the estimates. Assumptions of logistic regression were tested prior to the analyses, e.g., continuous determinants were categorized in case of a non-linear relationship with the outcome. To explore the non-responding group in more detail, we also imputed that group, and repeated the determinants analyses using multinomial regression analyses [21, 22].

Finally, we assessed the clinical implications of non-consent and non-response, studying CVRM measurement extractability in the EHR stratified by consent status as a proxy for the health care professional’s compliance to the CVRM guidelines. The data was considered extractable if it was registered in the designated field of the EHR.

R version 4.0.5. was used for all analyses [23].

## Results

### Patient characteristics

Out of the 5730 patients that were invited for participation between May 2015 and December 2019 (S4F1), 41.5% were consenting (*N* = 2378), 33.3% non-consenting (*N* = 1907) and 25.2% non-responding (*N* = 1445) (Table 1). Non-consenting patients were, compared to consenting patients, older, more often women, referred to the geriatric department, and had a higher cardiovascular burden: higher systolic blood pressure, higher 10-year CVD morbidity or mortality risk and more often a cardiovascular disease history. Non-responding patients were more physically active, had lower lipid values, less often a cardiovascular disease history and a lower 10-year CVD risk than both consenting and non-consenting patients. But please note that in the non-responding group, missing information was very considerable and limits the validity of these comparisons using the crude data.

**Table 1:** General characteristics stratified by consent status, based on the information extractable from the EHR

Variable	Total *N* = 5730	Consent *N* = 2378	Non-consent *N* = 1907	Non-response *N* = 1445
Age, median (Q1-Q3)^a^	67 (54.0–77.0)	63 (51.0–73.0)	71 (59.0–80.0)	66 (54.0–77.0)
Sex (women), N(%)^a^	2828 (49.4)	1083/2378 (45.5)	1025/1907 (53.7)	720/1445 (49.8)
High education, N(%)^b^	579 (19.2)	357/1724 (20.7)	153/1001 (15.3)	69/284 (24.3)
Current Smoking, N(%)^b^	471 (11.9)	264/2289 (11.5)	167/1281 (13.0)	40/372 (10.8)
Specialist specific OPD^a^				
-Cardiology	2783 (48.6)	1173/2378 (49.3)	815/1907 (42.7)	795/1445 (55.0)
-Diabetology	303 (5.3)	171/2378 (7.2)	60/1907 (3.1)	72/1445 (5.0)
-Geriatrics	1779 (31.0)	479/2378 (20.1)	909/1907 (47.7)	391/1445 (27.1)
-Nephrology	435 (7.6)	270/2378 (11.4)	74/1907 (3.9)	91/1445 (6.3)
-Vascular medicine	430 (7.5)	285/2378 (12.0)	49/1907 (2.6)	96/1445 (6.6)
Physical Activity per week (METminutes), median(Q1-Q3)^b^	4480.0 (2265.0- 7710.0)	4800 (2550.0–7995.0)	3675 (1638.8–6840.0)	5490.0 (2955.0–8026.5)
Previous AMI, CABG, CHF, Stroke, ICH, TIA, PAD (IC, AAA, Carotid), N(%)^b^	1400 (35.3)	782/2303 (34.0)	507/1292 (39.2)	111/372 (29.8)
Previous AMI, CABG, arrest, N(%)^b^	678 (17.1)	391/2303 (17.0)	226/1292 (17.5)	61/372 (16.4)
Previous CHF, N(%)^b^	401 (10.1)	237/2303 (10.3)	130/1292 (10.1)	34/372 (9.1)
Previous stroke, ICH, TIA, N(%)^b^	527 (13.3)	289/2303 (12.5)	207/1292 (16.0)	31/372 (8.3)
Previous peripheral arterial disease, N(%)^b^	364 (9.2)	196/2303 (8.5)	141/1292 (10.9)	27/372 (7.3)
Hypertension, N(%)^b^	1963 (49.5)	1126/2303 (48.9)	657/1292 (50.9)	180/372 (48.4)
Kidney disease, N(%)^b^	755 (19.0)	470/2303 (20.4)	230/1292 (17.8)	55/372 (14.8)
Proteinuria, N(%)^b^	521 (13.1)	344/2303 (14.9)	130/1292 (10.1)	47/372 (12.6)
Diabetes, N(%)^b^	833 (21.0)	504/2303 (21.9)	264/1292 (20.4)	65/372 (17.5)
Hyperlipidemia, N(%)^a^	1555 (43.4)	858/1945 (44.1)	588/1362 (43.2)	109/275 (39.6)
Body Mass Index (kg/m^2^), median(Q1-Q3)^a^	25.9 (23.1–29.4)	26.3 (23.5–29.6)	25.6 (22.9–29.1)	25.3 (22.6–28.7)
Heart rate (bpm), mean(SD)^a^	74.8 (14.6)	74.0 (14.1)	75.1 (14.7)	76.7 (15.8)
Systolic blood pressure (mmHg), mean(SD)^a^	141.7 (24.1)	140.7 (22.6)	145.1 (25.4)	134.6 (24.2)
Diastolic blood pressure (mmHg), mean(SD)^a^	80.0 (12.4)	80.8 (12.0)	80.3 (12.3)	75.4 (13.5)
Total Cholesterol (mmol/L), median(Q1-Q3)^a^	5.1 (4.2–6.0)	5.1 (4.3–6.0)	5.1 (4.2–6.0)	4.8 (3.9–5.8)
HDL-Cholesterol (mmol/L), median(Q1-Q3)^a^	1.3 (1.1–1.6)	1.3 (1.1–1.6)	1.3 (1.1–1.6)	1.2 (1.0–1.5)
Hb (mmol/L), mean(SD)^a^	8.6 (1.1)	8.8 (1.0)	8.5 (1.1)	8.1 (1.3)
Creatinine (μmol/L), median(Q1-Q3)^a^	76.0 (64.0–95.0)	76 (65.0–93.0)	75 (63.0–94.0)	79 (65.0–102.8)
eGFR (CKD epi), mean(SD)^a^	77.5 (25.9)	80.1 (24.9)	75.7 (25.5)	73.7 (28.6)
HbA1c (mmol/mol), median(Q1-Q3)^a^	38.0 (35.0–44.0)	38 (35.0–43.0)	38 (35.0–44.0)	41 (36.0–52.0)
10-yr risk on CVD, median(Q1-Q3)^c^	10.0 (3.0–28.0)	9.0 (3.0–24.6)	12.7 (4.0–29.9)	8.0 (5.0–18.5)

*Notes:* ^a^= routine care data from the EHR; ^b^= data from the UCC-CVRM questionnaire; ^c^= calculated based on routine care and questionnaire data; *N* number, *Q1* first quartile, *Q3* third quartile, % percentage, *OPD* Outpatient department, *MET* Metabolic Equivalent of Task, *AMI* Acute myocardial infarction, *CABG* Coronary artery bypass grafting, *CHF* Congestive heart failure, *ICH* Intracranial hemorrhage, *TIA* Transient ischemic attack, *PAD* Peripheral arterial disease, *AAA* Abdominal aortic aneurysm, *bpm* Beats per minute, *SD* Standard deviation, *HDL* High-density lipoprotein, *Hb* Haemoglobin, *eGFR* Estimated glomerular filtration rate using the CKD-EPI formula, *CKD* Chronic kidney disease, *HbA1c* glycated haemoglobin, *yr* year

### Age and treating specialty stratified by sex

**Fig. 1:**
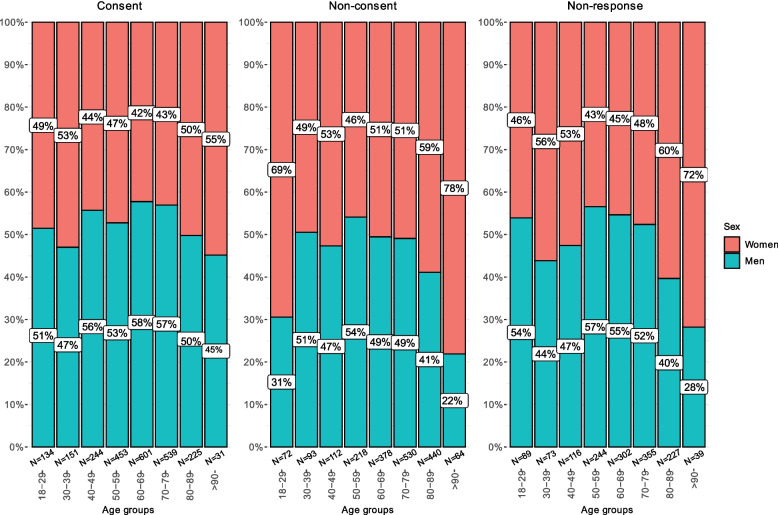
Sex distribution within age categories across consent groups

The non-consent group had the highest percentage of patients aged 70 years or older (54.3%) compared to 33.4% of the consent-group and 43.0% of the non-response group. In the non-consent group, most patients in the lowest and the two highest age groups were women (69%, 59% and 78%, respectively, Fig. 1). We performed sensitivity analyses to investigate whether the differences in the age distribution between the consent, non-consent and non-response group could be related to the high number of patients referred to the geriatric department. These analyses illustrated that the geriatrics department had less variability in age than the other departments and that their age was considerable higher (S5F1). Analyses excluding the geriatric department showed that the age differences between the consent and non-consent group declined (S5T1).

**Fig. 2:**
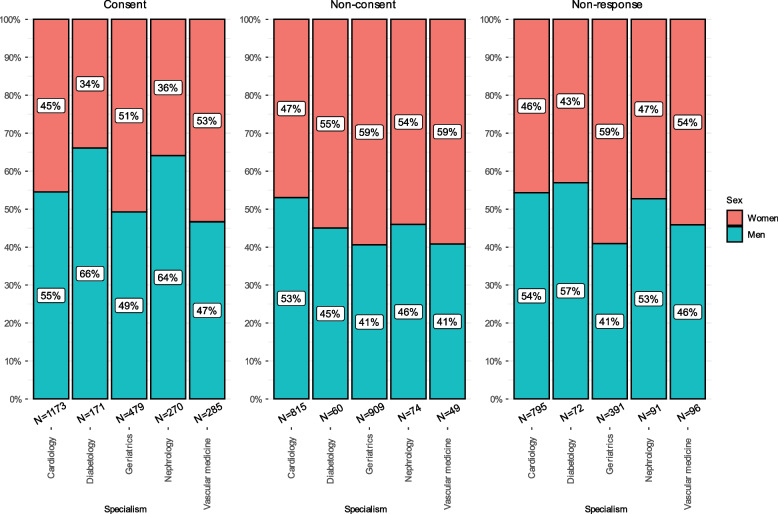
Sex distribution within treating specialties across consent groups

The percentage of consenting, non-consenting, and non-responding patients varied across medical specialties (Fig. 2). Geriatrics accounted for almost half of all non-consenting patients (47.7%). Geriatrics and vascular medicine were least equally distributed in terms of sex in the non-consent group (18% more women than men).

### Determinants of non-consent

**Fig. 3:**
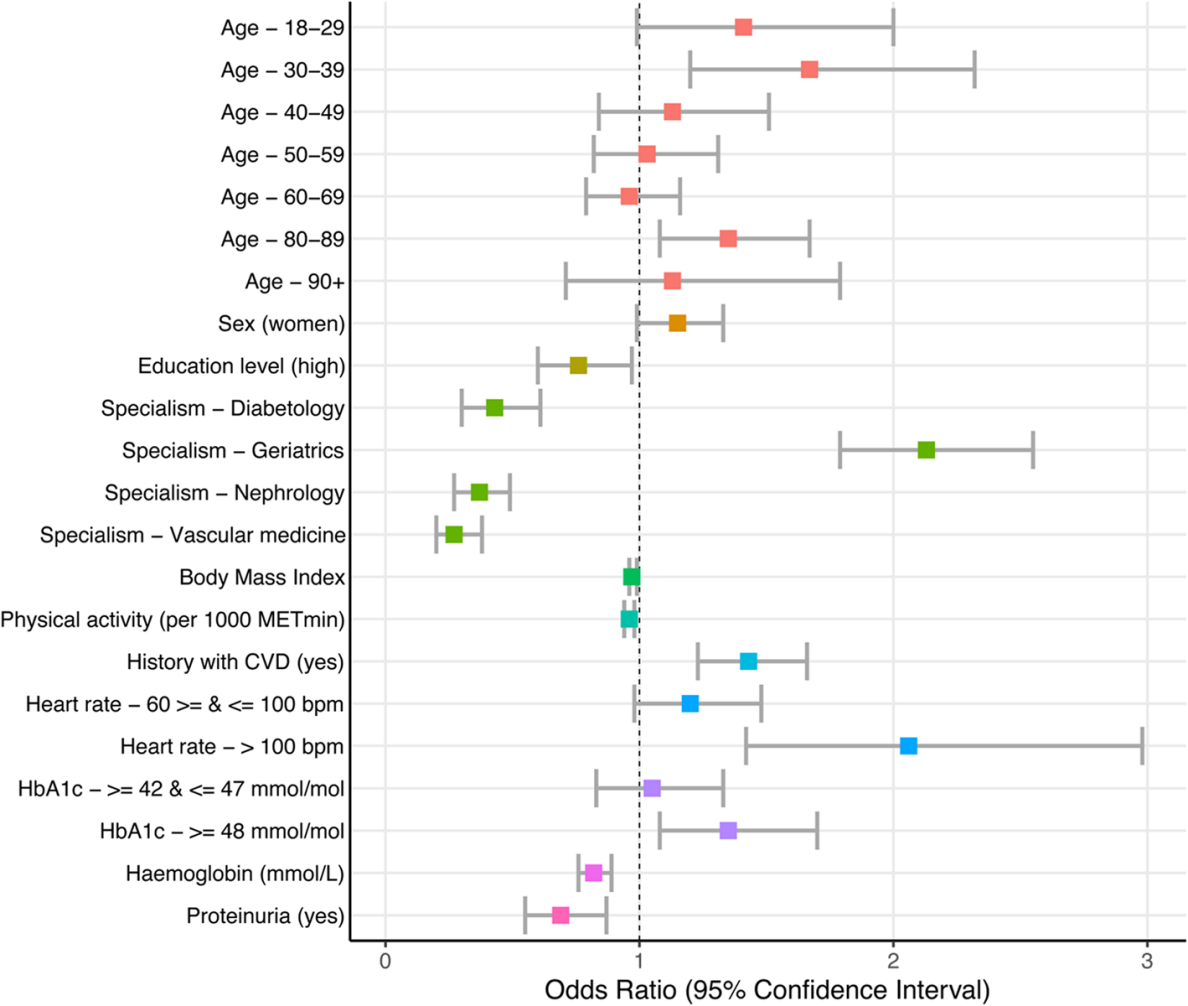
Determinants associated with non-consent compared to consent, including 95% confidence intervals. METmin = metabolic equivalent of task-minutes; CVD = cardiovascular diseases; bpm = beats per minute; HbA1c = glycated haemoglobin; OR = odds ratio indicating the likelihood of having provided a non-consent as compared to a consent; % = percentage

Results from the multivariable logistic regression model showed that patients with increased odds of non-consent were being referred to the geriatric department (OR 2.13 (1.79–2.55)), aged between 30–39 or 80–89 years old, more often had a heartrate > 100 bpm (OR 2.06 (1.42–2.98)), HbA1c levels > 48 mmol/mol (OR 1.35 (1.08–1.70)) and a history of cardiovascular diseases (OR 1.43 (1.23–1.66)) (Fig. 3, S6T1). Furthermore, patients with lower odds of non-consent had a high education level (OR 0.76 (0.60–0.97)), proteinuria (OR 0.69 (0.55–0.87)), and higher BMI and physical activity levels. Additionally, being referred to the vascular medicine, nephrology and diabetology outpatient clinic decreased the odds of non-consent compared to the cardiology outpatient clinic.

### Exploratory analyses: determinants of non-response

The associations related to non-consent and non-response followed a similar pattern regarding age, heamoglobin, heartrate, BMI, and cardiovascular disease history (S7F1). Different was that a high education level, high physical activity level, peripheral arterial disease history, HbA1c level >  = 42 mmol/mol and low blood pressure were more strongly related to non-response compared to the associations found related to non-consent. Being referred to the geriatrics department and proteinuria, however, were not significantly associated with non-response.

### Clinical implications of non-consent and non-response

Registration of extractable CVRM data from the EHR was 5 to 10% lower in non-consenting patients compared to consenting patients (Table 2). Moreover, availability reduced by more than 25% in items that were extracted from the UCC-CVRM questionnaire (history of CVD, smoking status, and hypertension history). Because of the impossibility of extracting such data form the EHR, 10-year risk estimates on cardiovascular morbidity or mortality were only available in 41% of non-consenting patients. The extractability of CVRM data in the non-response group was lower, ranging between the 2% and 50%.

**Table 2:** Extractability of the CVRM data in % in the EHR, stratified by consent status

	Consent	Non-consent	Non-response
BMI present (%)	94	88	48
SBP present (%)	94	89	35
DBP present (%)	94	89	35
Smoking status present (%)	96	67	26
CVD history present (%)	97	68	26
Hypertension history present (%)	97	68	26
Creatinine present (%)	87	82	50
Total cholesterol present (%)	83	73	20
HDL-c present (%)	82	72	19
Hb present (%)	87	82	50
CVD 10-yr risk estimation present(%)	71	41	2

*Notes: BMI* Body Mass Index, % percentage, *SBP* Systolic blood pressure, *DBP* Diastolic blood pressure, *CVD* Cardiovascular disease, *HDL-c* High-density lipoprotein cholesterol, *Hb* Haemoglobin, *yr* year

## Discussion

### Summary of the findings

We studied the differences between non-consenting, non-responding and consenting patients. Furthermore, we assessed whether cardiovascular risk indicator extractability from the EHR differed between the consent categories. We found that patients who were between 30–39 or 80–89 years old, less physically active, less educated, with a higher heartrate, higher HbA1c levels, lower haemoglobin levels, and a cardiovascular disease history were more often non-consenting. Non-response was associated with an age between 18–29 and 80–89, high education level, high physical activity level, low BMI, cardiovascular disease history, a high heart rate, low haemoglobin levels, low systolic blood pressure and higher HbA1c- levels.

### Comparison with the literature

To our knowledge limited research has been conducted on the characteristics of non-consenting and non-responding patients, especially in cardiovascular LHSs targeting patients that are referred to the hospital for secondary prevention. Previous research on non-consent and, more often, non-response was mostly conducted in population-based research settings and clinical trials and not necessarily related to cardiovascular disease, severely limiting a good comparison between our results and the existing literature.

However, these studies found that non-consenting and non-responding patients generally were less educated [24–27], older [24, 28], women [28, 29], less physically active [30], more smokers [28], and had a higher disease burden [24, 28, 31, 32]. On the contrary, others reported non-responders to be younger [29, 31, 33].

Our findings regarding non-consent can partially be explained by the ‘worried well’ phenomenon: relatively healthy (younger) and educated patients tend to seek more medical advice and are more willing to participate in studies [26, 34, 35]. Participating in the UCC-CVRM LHS may give these patients the sense that their health is more closely monitored. An important addition from our study is that non-consent rates varied considerably across departments to which the patients were referred to for evaluation. We found that most of the non-consenting patients were referred to the geriatrics department and that they were older than the patients referred to the other departments. The elderly population is a heterogeneous group consisting of vital and frail elderly patients. Compared to more vital elderly patients, frail elderly patients are more at-risk for negative health outcomes [36]. The geriatrics department will be visited more by frail elderly patients, whereas other outpatient clinics will more likely be visited by more vital elderly patients. Previous research showed that frail elderly patients are more difficult to include into clinical studies because of the challenges associated with obtaining informed consent from this group [37, 38].

In addition, previous studies showed education level, among others, to be associated with health literacy [39, 40]. Others found that limited health literacy was associated with limited understanding of consent forms, leading to anxiety and less satisfaction with the consent process [41]. Clinical trial consent forms often appear to be written in an above average health literacy level, which, as a consequence, means that patients do not understand what they could be consenting for and, therefore, decline the invitation to participate [42]. Although the UCC-CVRM is not a clinical trial and our ethics committee demands writing patient information on a last class primary school level, this could be an explanation for the non-consenting patients more often being less educated.

The ‘worried-well’ phenomenon would, however, not entirely justify why patients with higher BMI and a history of proteinuria were at lower risk of non-consent. It is known that BMI changes with age and that the pattern of change is U-shaped. BMI increases in the younger age groups and then decreases in the older age groups [43, 44]. With the non-consent group being older, it is evident that our results point towards an association between lower BMI and non-consent. Nevertheless, generally healthy patients with an increased BMI could also be worried about their health and, therefore, agree to participate in a cardiovascular LHS. Data on proteinuria, however, was extracted from the self-reported UCC-CVRM questionnaire. The literal question was: *“Have you ever been diagnosed with protein in your urine?”* and, thus, not specifically stating *increased* protein levels in their urine, potentially leading patients with a normal amount of protein in their urine to answer this question with ‘yes’.

Surprising and in contrast to the associations found in the multivariable logistic regression analysis regarding the determinants of non-consent was the finding that a high education and physical activity level was associated with non-response. This might suggest that different profiles of patients are combined in the non-responding group: the patients with a condition already regularly controlled and the younger healthier group. These results should, however, be interpreted cautiously due to the significant missingness in the non-response group, potentially hampering the validity of these results. Nevertheless, and in line with previous research, non-responding patients seemed different from the consenting patients, but also from non-consenting patients [10, 27]. Indicating that, when studying non-participation, one should consider keeping the different categories of non-participation separate, even though non-response and non-consent are often considered to be the same.

### Informed consent, participation bias on causal research

Our study indicates that an informed consent procedure in a cardiovascular LHS leads to a misrepresentation of the target population by the consenting patients, hampering generalisability of research results. We did, however, not assess whether this led to over -or underestimation of the associations between cardiovascular risk indicators in the target population compared to the associations within consenting patients. We recommend this for future research.

### Informed consent and impact on learning healthcare systems

Routine clinical care data is increasingly used to improve the quality of care in a LHS approach as well as for research purposes. However, because of the apparent participation bias, the validity and generalisability of the results from research based on routine care data of the LHS could be at stake.

There is not yet a clear approach as to how patients should consent for the use of their routine clinical care data in a LHS design, as there is no consensus about the requirement of an informed consent [45]. However, a traditional informed consent procedure does not seem to be fit for purpose. The concerns about bias as a result of the traditional informed consent procedure in cohort studies have been expressed before [46]. Cumyn et al. [45] reviewed different types of consent forms, indicating meta-consent or dynamic consent as being the most appropriate within a LHS. Additionally, they emphasize the importance of information transfer between the professional and the patient. When communication about the LHS is lacking, patients are less likely to consent for the use of their data [45].

### Impact on clinical care

Previous research from Groenhof et al. [47] compared the completeness of extractable CVRM indicators before and after the UCC-CVRM initiation in consenting patients. They showed that an infrastructure such as the UCC-CVRM LHS leads to a substantial improvement in the completeness of these indicators in the EHR, further enabling the use of, among others, cardiovascular risk algorithms, resulting in more information on the patient’s cardiovascular risk profile when determining the treatment strategy [47]. However, some patients in our study actively refused to participate in the LHS or did not respond to the invitation, which may have led to the less structured registration of CVRM indicators found in our study. This might affect the optimal CVRM in routine practice.

## Conclusion

An informed consent procedure in a LHS may lead to participation bias. Furthermore, structured registration of CVRM indicators in the EHR was less in non-consenting and non-responding patients, which is detrimental for the LHS feedback loop, and potentially leads to suboptimal CVRM. This study underlines the importance of reassessing the need of a traditional informed consent procedure for the use of routine clinical care data in LHSs.

## Supplementary Information

**Additional file 1:** **Supplement 1.** STROBE checklist for reporting. **Supplement 2.** UCC-CVRM questionnaire. **Supplement 3.** Missingness. **Supplement 4.** Patient inclusion flow-chart. **Supplement 5.** Sensitivity analysis to explore age distributions. **Supplement 6.** Determinants of non-consent. **Supplement 7.** Determinants of non-response (exploratory analysis).

## Data Availability

The dataset analysed during the current study is not publicly available due to privacy and ethical concerns but is available from the corresponding author on reasonable request.

## Abbreviations

LHS: Learning Healthcare System
CVRM: Cardiovascular riskmanagement
UMC: University Medical Center
BMI: Body Mass Index
UCC: Utrecht Cardiovascular Cohort
EHR: Electronic Health Record
LDL: Low-Density Lipoprotein
MET: Metabolic Equivalent of Task
SQUASH: Short QUestionnaire To ASsess Health-enhancing Physical Activity
CVD: Cardiovascular Disease
SCORE: Systematic COronary Risk Evaluation
UKPDS: United Kingdom Prospective Diabetes Study
SMART: Second Manifestations of Arterial Disease
COVID: Coronavirus Disease
ANOVA: Analysis of Variance
MICE: Multivariate Imputation by Chained Equations
OPD: Outpatient Department
AMI: Acute Myocardial Infarction
CAGB: Coronary Artery Bypass Grafting
CHF: Congestive Heart Failure
ICH: Intracranial Hemorrhage
TIA: Transient Ischemic Attack
PAD: Peripheral Arterial Disease
AAA: Abdominal Aortic Aneurysm
HDL: High-Density Lipoprotein
Hb: Haemoglobin
eGFR: Estimated Glomerular Filtration Rate
CKD: Chronic Kidney Disease
HbA1c: Glycated haemoglobin
OR: Odds Ratio
CI: Confidence Interval
SBP: Systolic blood pressure
DBP: Diastolic blood pressure
