# Supplementary material for: Asking informed consent may lead to significant participation bias and suboptimal cardiovascular risk management in learning healthcare systems
Source: BMC Med Res Methodol. 2023 Apr 22;23:98. doi: 10.1186/s12874-023-01924-6 (PMC10122202; doi:10.1186/s12874-023-01924-6)
Supplement: Supplementary file 1 — Additional file 1: Supplement 1. STROBE checklist for reporting. Supplement 2. UCC-CVRM questionnaire. Supplement 3. Missingness. Supplement 4. Patient inclusion flow-chart. Supplement 5. Sensitivity analysis to explore age distributions. Supplement 6. Determinants of non-consent. Supplement 7. Determinants of non-response (exploratory analysis). [file 12874_2023_1924_MOESM1_ESM.docx]

**Supplementary Material**

**Supplement 1 – STROBE checklist for reporting**

**S1T1. STROBE checklist for cross-sectional studies.**

|  | Item No | Recommendation | Page No |
| --- | --- | --- | --- |
| **Title and abstract** | 1 | (*a*) Indicate the study’s design with a commonly used term in the title or the abstract | 1 |
|  |  | (*b*) Provide in the abstract an informative and balanced summary of what was done and what was found | 2 |
| Introduction | | | |
| Background/rationale | 2 | Explain the scientific background and rationale for the investigation being reported | 3,4 |
| Objectives | 3 | State specific objectives, including any prespecified hypotheses | 4 |
| Methods | | | |
| Study design | 4 | Present key elements of study design early in the paper | 5 |
| Setting | 5 | Describe the setting, locations, and relevant dates, including periods of recruitment, exposure, follow-up, and data collection | 5 |
| Participants | 6 | (*a*) Give the eligibility criteria, and the sources and methods of selection of participants | 5,6 |
| Variables | 7 | Clearly define all outcomes, exposures, predictors, potential confounders, and effect modifiers. Give diagnostic criteria, if applicable | 6,7 |
| Data sources/ measurement | 8* | For each variable of interest, give sources of data and details of methods of assessment (measurement). Describe comparability of assessment methods if there is more than one group | 6,7 |
| Bias | 9 | Describe any efforts to address potential sources of bias | 7,17 |
| Study size | 10 | Explain how the study size was arrived at | NA |
| Quantitative variables | 11 | Explain how quantitative variables were handled in the analyses. If applicable, describe which groupings were chosen and why | 6,7 |
| Statistical methods | 12 | (*a*) Describe all statistical methods, including those used to control for confounding | 7 |
|  |  | (*b*) Describe any methods used to examine subgroups and interactions | 7 |
|  |  | (*c*) Explain how missing data were addressed | 7 |
|  |  | (*d*) If applicable, describe analytical methods taking account of sampling strategy | NA |
|  |  | (*e*) Describe any sensitivity analyses | 10 |

| Results | | | |
| --- | --- | --- | --- |
| Participants | 13* | (a) Report numbers of individuals at each stage of study—eg numbers potentially eligible, examined for eligibility, confirmed eligible, included in the study, completing follow-up, and analysed | 8, S4F1 |
|  |  | (b) Give reasons for non-participation at each stage | S4F1 |
|  |  | (c) Consider use of a flow diagram | S4F1 |
| Descriptive data | 14* | (a) Give characteristics of study participants (eg demographic, clinical, social) and information on exposures and potential confounders | 8,9 |
|  |  | (b) Indicate number of participants with missing data for each variable of interest | 9, S3T1 |
| Outcome data | 15* | Report numbers of outcome events or summary measures | 8-13 |
| Main results | 16 | (a) Give unadjusted estimates and, if applicable, confounder-adjusted estimates and their precision (eg, 95% confidence interval). Make clear which confounders were adjusted for and why they were included | 11-13 |
|  |  | (b) Report category boundaries when continuous variables were categorized | 10-13 |
|  |  | (c) If relevant, consider translating estimates of relative risk into absolute risk for a meaningful time period | NA |
| Other analyses | 17 | Report other analyses done—eg analyses of subgroups and interactions, and sensitivity analyses | 10, S5F1, S5T1 |
| Discussion | | | |
| Key results | 18 | Summarise key results with reference to study objectives | 14 |
| Limitations | 19 | Discuss limitations of the study, taking into account sources of potential bias or imprecision. Discuss both direction and magnitude of any potential bias | 16 |
| Interpretation | 20 | Give a cautious overall interpretation of results considering objectives, limitations, multiplicity of analyses, results from similar studies, and other relevant evidence | 14-17 |
| Generalisability | 21 | Discuss the generalisability (external validity) of the study results | 16 |
| Other information | | | |
| Funding | 22 | Give the source of funding and the role of the funders for the present study and, if applicable, for the original study on which the present article is based | 19 |

**Supplement 2 - UCC-CVRM questionnaire**

All patients that were referred to the UMC Utrecht for a first time evaluation of a cardiovascular disease or risk factor and, thus, eligible for UCC-CVRM, were asked to fill in this questionnaire (S2).

**Personal details**

Initials and name: _______________________________________________________________________ M / F

Residence: _______________________________________________________________________

Date of birth (day/month/year): _______________ / _______________ / _______________

Email address (if available) _______________________________________________________________________

Date of (day/month/year): _______________ / _______________ / _______________

Department you are visiting: _______________________________________________________________________

**1. Length, weight, education**

**1.01** What’s your height? _______________ cm

**1.02** What’s your bodyweight? _______________ kg

- 1. What is the highest level of education you have completed?
- Primary school/ no education
- Preparatory secondary vocational education
- Senior general secondary education or university preparatory education
- Lower professional education
- Secondary vocational education
- University of applied sciences
- University
- Other, please specify:_______________________________________________________________________________

**2. Smoking**

**2.01** Do you smoke? (cigarettes/ tobacco)

- Yes
- No ☞***continue with question 2.04***
  1. How many cigarettes do you smoke on average each day? _______________ (number)

**2.03** Which year did you commence smoking? ___________ Year)

☞***continue with question 3***

**2.04** Did you **ever** smoke (cigarettes/tobacco) ?

- Yes
- No ☞ ***continue with question 3***

**2.05** How many cigarettes did you smoke on average each day _______________ (number)

**2.06** When did you start smoking? ___________ (year)

**2.07** When did you quit smoking? ___________ (year)

**3. Medical history**

**3.01** Were you ever diagnosed with a heart attack (heart infarction/ myocardial infarction)?

(This does not include cardiac arrest)

- Yes

When were you diagnosed with a (if multiple, please report the first) heart attack? ___________(year)

- No
- I don’t know

**3.02** Have you ever had treatment for a narrowed coronary artery(/ies). Coronary arteries are the arteries that supply the heart with oxygen rich blood.

(Percutaneous transluminal coronary angioplasty (PTCA), stenting, open heart surgery with bypasses)

- Yes

When did you have this treatment (if multiple, please report the first) ? ____________(year)

- No
- I don’t know

**3.03** Did you ever experience a cardiac arrest?

(During a cardiac arrest the heart does not beat at all)

- Yes

When did you experience your (if multiple, please report the first) cardiac arrest ____________(year)

- No
- I don’t know

**3.04** Were you ever diagnosed with heart failure?

(Heart failure is characterized by a diminished pump function of the heart)

- Yes

When were you diagnosed with heart failure? ____________(year)

- No
- I don’t know

**3.05** Were you ever diagnosed with atrial fibrillation?

(Atrial fibrillation abnormal heart rhythm characterized by rapid and irregular beating of the heart)

- Yes

When were you diagnosed with atrial fibrillation? ____________(year)

- No
- I don’t know

**3.06** Were you ever diagnosed with a cerebral infarction (ischemic stroke)?

(A cerebral infraction is characterized by a disruption of the blood flow to the brain. Common symptoms are face drooping, abnormal speech and paralyses of limbs.)

- Yes

When were you diagnosed with a (if multiple, please report the first) stroke? ____________(year)

- No
- I don’t know

**3.07** Were you ever diagnosed with a brain bleed (cerebral hemorrhage)?

(A cerebral hemorrhage is a bleed within the brain caused by the rupture of a vessel within the brain.)

- Yes

When were you diagnosed with a (if multiple, please report the first) brain bleed? ____________(year)

- No
- *I don’t know*

**3.08** Did you ever experience a TIA?

(During a TIA – transient ischemic attack – there is a **temporary** disruption of the blood flow to the brain. Common symptoms are face drooping, abnormal speech and paralyses of limbs. These symptoms resolve within 24 hours without medical intervention)

- Yes

When did you experience your (if multiple, please report the first) TIA? ____________(year)

- No
- I don’t know

**3.09** Have you ever had invasive treatment for a narrowed carotid artery?

(The carotid artery is one of the largest arteries in your neck. This treatment may be either an operation, stenting or balloon dilatation)

- Yes

When did you have this treatment (if multiple, please report the first time) ? ____________(year)

- No
- I don’t know

**3. 10** Did you ever experience intermitted claudication/peripheral vascular disease?

(Peripheral vascular disease is vascular damage of the vessels in the limbs)

- Yes

When were you diagnosed with atrial fibrillation? ____________(year)

- No
- I don’t know

**3.11** Have you ever had invasive treatment for narrowed leg arteries?

(This treatment can be either an operation, stenting or balloon dilatation)

- Yes

When did you have this treatment (if multiple, please report the first time) ? ____________(year)

- No
- I don’t know

**3.12** Have you ever had invasive treatment for an aneurysm or narrowing of your abdominal aorta?

(This treatment may be either an operation, stenting or balloon dilatation)

- Yes

When did you have this treatment (if multiple, please report the first time)? ____________(year)

- No
- I don’t know

**3.13** Were you ever diagnosed with high blood pressure?

- Yes

When were you diagnosed with atrial fibrillation? ____________(year)

- No
- I don’t know

**3.14** Were you ever diagnosed with renal failure?

(Renal failure is when the kidneys do not function well)

- Yes

When were you diagnosed with atrial fibrillation? ____________(year)

- No
- I don’t know

**3.15** Were you ever diagnosed with proteinuria (proteins in the urine)?

(This does not include proteinuria during pregnancy)

- Yes

When were you diagnosed with proteinuria? ____________(year)

- No
- I don’t know

**3.16** Are you a diabetic?

(This does not include gestational diabetes, which is diabetes that only occurs during pregnancy)

- Yes

When were you diagnosed with diabetes? ____________(year)

- No
- I don’t know

**3.17** Were you ever diagnosed with high cholesterol (dyslipidemia/hypercholesterolemia)?

- Yes

When were you diagnosed with high cholesterol? ____________(year)

- No
- I don’t know

**4. Medication**

**4.01** Are you using any medications?

- Yes, please take a list of all your medications to your doctor.
- No

**4.02** Please state which medications you use, what dosage, which frequency and since when

**Name medication Dosage Times per day Since (Year**)

*Example: Zocor 20 mg 1 time 1999*

1. ______________________________________________ ­­­­_________________ _____________________
2. ______________________________________________ ­­­­_________________ _____________________
3. ______________________________________________ ­­­­_________________ _____________________
4. ______________________________________________ ­­­­_________________ _____________________
5. ______________________________________________ ­­­­_________________ _____________________
6. ______________________________________________ ­­­­_________________ _____________________
7. ______________________________________________ ­­­­_________________ _____________________
8. ______________________________________________ ­­­­_________________ _____________________
9. ______________________________________________ ­­­­_________________ _____________________
10. ______________________________________________ ­­­­_________________ _____________________
11. ______________________________________________ ­­­­_________________ _____________________
12. ______________________________________________ ­­­­_________________ _____________________

**5. Alcohol**

**5.01** Do you drink any alcoholic beverage?

- Yes
- No ☞ ***continue with question 5.04***

**5.02** How many glasses of alcoholic beverages do you drink on average per **week**? ______________ (number)

**5.03** When did you start drinking alcoholic beverages? _______________ (year)

**5.04** Did you **ever** consume any alcoholic beverages?

- Yes
- No ☞ ***continue with question 6***

**5.05** How many glasses of alcoholic beverages did you drink on average per **week**? ______________ (number)?

**5.06** When did you start drinking alcoholic beverages? _______________ (year)

**5.07** When did you quit drinking alcoholic beverages? ____________ (year)

**6. Sports and physical activity**

**6.01** Please report on an average week during the last few months.

Would you please report how many year per week you would do the below mentioned activity, how many minutes on average and how vigorous the activity was

| **Commuting to work/school** | **Number of days**  **per *week*** | **Average time**  **per *day*** | | **Vigorosity**  **(circle your answer)** | |
| --- | --- | --- | --- | --- | --- |
| Waling to/from work or school  Cycling to/ from work or school  Not applicable | days  days | hours minutes  hours minutes | | slow/ intermediate/ fast  slow/ intermediate/ fast | |
| **Physical activity at work/school** | | | | **Average time**  **per week** | |
| Light/intermediate vigorous work (sitting, now and then walking  Heavy duty/intensely vigorous work  Not applicable | | | | hours minutes  hours minutes | |
| **Household activities** | | **Number of days**  **per *week*** | | **Average time**  **per *day*** | |
| Light/intermediate vigorous work (ironing, feeding a child, etc.)  Heavy duty/intensely vigorous household work  Not applicable | | days  days | | hours minutes  hours minutes | |
| **Spare time** | **Number of days**  **per *week*** | **Average time**  **per *day*** | | **Vigorosity**  **(circle your answer)** | |
| Walking  Cycling  Gardening  Jobs around the house /DIY  Not applicable | days  days  days  days | hours minutes  hours minutes  hours minutes  hours minutes | | | slow /intermediate/ fast  slow/ intermediate/ fast  light/ intermediate/ heavy  light / intermediate/ heavy |
| **Exercise/sports** (write down maximum of 4)  *Example: tennis, soccer, swimming… etc.* | | |  | **Vigorosity**  **(circle your answer)** | |
| 1. ...........................................  2. ...........................................  3. ...........................................  4. ...........................................  Not applicable | days  days  days  days | hours minutes    hours minutes  hours minutes  hours minutes | | | light / intermediate/ heavy  light / intermediate/ heavy  light / intermediate/ heavy  light / intermediate/ heavy |
| **TOTAL**  On average, how many days a week do you spent at least 30 minutes a day with walking, cycling, jobs around the house, gardening or sports? | | | |  | |
|  |  |  |  | days per week | |

**7. Family history**

Cardiovascular diseases partly have a genetic origin. That is why we would like to have information about your family and the possible presence of cardiovascular disease in your family. With family we mean parents, brothers and sisters, and your children.

**7.01** Your country of birth: _______________________________________________________________________

**7.02** Country of birth father: _______________________________________________________________

**7.03** What is the highest level of education your father has completed?

- Primary school/ no education
- Preparatory secondary vocational education
- Senior general secondary education or university preparatory education
- Lower professional education
- Secondary vocational education
- University of applied sciences
- University
- Other, please specify:_______________________________________________________________________________

**7.04** Country of birth mother: _____________________________________________________________

**7.05** What is the highest level of education your mother has completed?

- Primary school/ no education
- Preparatory secondary vocational education
- Senior general secondary education or university preparatory education
- Lower professional education
- Secondary vocational education
- University of applied sciences
- University
- Other, please specify:_______________________________________________________________________________

**7.06** How many brothers do you have? ________________________________________________________________

**7.07** How many sisters do you have? _______________________________________________________________

**7.08** How many sons do you have? ________________________________________________________________

**7.09** How many daughters do you have? _____________________________________________________________

**7.10** Please state the year of birth of your father, mother and any brother (s), sister (s) and child (ren) in the column for birth year.

If your father, mother, possible brother (s) and sister (s) experienced one of the diseases or causes of death stated below, please indicate this by writing down the age of occurrence in that cell within the table

*Example:*

*Your* ***father*** *was born in* ***1891*** *and had a* ***heart*** ***attack*** *at the age of* ***88****.*

*Your* ***mother*** *was born in* ***1899*** *and has had* ***none*** *of the aforementioned condition.*

*You have a* ***brother*** *who was born in* ***1920*** *and had a* ***cerebral*** ***infarction*** *at* ***56*** *years of age.*

*You would fill in the table as followed:*

|  | **Father** | **Mother** | **Brother 1** | **Brother 2** |
| --- | --- | --- | --- | --- |
| **Year of birth** | 1891 | 1899 | 1920 |  |
| **Heart attack + age** | 88 |  |  |  |
| **Cerebral hemorrhage + age** |  |  | 56 |  |

|  | **Father** | **Mother** | **Brother 1** | **Brother 2** | **Brother 3** | **Brother 4** | **Brother 5** | **Sister 1** | **Sister 2** | **Sister 3** | **Sister 4** | **Sister 5** | **Son 1** | **Son 2** | **Son 3** | **Son 4** | **Son 5** | **Daughter 1** | **Daughter 2** | **Daughter 3** | **Daughter 4** | **Daughter 5** |
| --- | --- | --- | --- | --- | --- | --- | --- | --- | --- | --- | --- | --- | --- | --- | --- | --- | --- | --- | --- | --- | --- | --- |
| **Year of birth** |  |  |  |  |  |  |  |  |  |  |  |  |  |  |  |  |  |  |  |  |  |  |
| **No information available** |  |  |  |  |  |  |  |  |  |  |  |  |  |  |  |  |  |  |  |  |  |  |
| **Brain bleed**  **+ age** |  |  |  |  |  |  |  |  |  |  |  |  |  |  |  |  |  |  |  |  |  |  |
| **Cerebral infarction**  **+ age** |  |  |  |  |  |  |  |  |  |  |  |  |  |  |  |  |  |  |  |  |  |  |
| **Narrowed carotid arteries**  **+ age** |  |  |  |  |  |  |  |  |  |  |  |  |  |  |  |  |  |  |  |  |  |  |
| **Heart attack**  **+ age** |  |  |  |  |  |  |  |  |  |  |  |  |  |  |  |  |  |  |  |  |  |  |
| **Heart surgery**  **+ age** |  |  |  |  |  |  |  |  |  |  |  |  |  |  |  |  |  |  |  |  |  |  |
| **Heart failure**  **+ age** |  |  |  |  |  |  |  |  |  |  |  |  |  |  |  |  |  |  |  |  |  |  |
| **Narrowed carotid arteries**  **+ age** |  |  |  |  |  |  |  |  |  |  |  |  |  |  |  |  |  |  |  |  |  |  |
| **Aneurysm of abdominal aorta**  **+ age** |  |  |  |  |  |  |  |  |  |  |  |  |  |  |  |  |  |  |  |  |  |  |
| **Narrowed vessels in limbs**  **+ age** |  |  |  |  |  |  |  |  |  |  |  |  |  |  |  |  |  |  |  |  |  |  |
| **Cause of death: brain bleed**  **+ age** |  |  |  |  |  |  |  |  |  |  |  |  |  |  |  |  |  |  |  |  |  |  |
| **Cause of death: cerebral infarction**  **+ age** |  |  |  |  |  |  |  |  |  |  |  |  |  |  |  |  |  |  |  |  |  |  |
| **Cause of death: heart attack**  **+ age** |  |  |  |  |  |  |  |  |  |  |  |  |  |  |  |  |  |  |  |  |  |  |
| **Sudden death (no accident)**  **+ age** |  |  |  |  |  |  |  |  |  |  |  |  |  |  |  |  |  |  |  |  |  |  |
| **Cause of death: other**  **+ age** |  |  |  |  |  |  |  |  |  |  |  |  |  |  |  |  |  |  |  |  |  |  |

The next questions are only applicable to women. Are you a man? ☞ ***this is the end of the questionnaire***

**8. Pregnancy**

**8.01** Are you pregnant (currently)?

- Yes
- No

**8.02** Were you ever pregnant?

- Yes
- No ☞ ***this is the end of the questionnaire***

**8.03** How many pregnancies did you experience (miscarriage included) ___________ (number)

**8.04** The next question is about the course of your pregnancies:

| Pregnancy 🡪 | 1 | | 2 | | 3 | 4 | | | 5 | | 6 |
| --- | --- | --- | --- | --- | --- | --- | --- | --- | --- | --- | --- |
| How did you become pregnant? | Spontaneous  IUI  IVF  ICSI  Other | | Spontaneous  IUI  IVF  ICSI  Other | | Spontaneous  IUI  IVF  ICSI  Other | Spontaneous  IUI  IVF  ICSI  Other | | | Spontaneous  IUI  IVF  ICSI  Other | | Spontaneous  IUI  IVF  ICSI  Other |
| Pregnancy outcome  *extra uterine pregnancy is when the fetus grows somewhere in the body outside the womb | Vaginal birth  Cesarean section  Miscarriage  Abortion  Extra-uterine pregnancy * | | Vaginal birth  Cesarean section  Miscarriage  Abortion  Extra-uterine pregnancy * | | Vaginal birth  Cesarean section  Miscarriage  Abortion  Extra-uterine pregnancy * | Vaginal birth  Cesarean section  Miscarriage  Abortion  Extra-uterine pregnancy * | | | Vaginal birth  Cesarean section  Miscarriage  Abortion  Extra-uterine pregnancy * | | Vaginal birth  Cesarean section  Miscarriage  Abortion  Extra-uterine pregnancy * |
| Total weeks of pregnancy |  | |  | |  |  | | |  | |  |
| Year of the end of the pregnancy |  | |  | |  |  | | |  | |  |
| Your age during this pregnancy |  | |  | |  |  | | |  | |  |
| Sex child | boy  girl  Unknown | | boy  girl  Unknown | | boy  girl  Unknown | boy  girl  Unknown | | | boy  girl  Unknown | | boy  girl  Unknown |
| Birthweight child | ___________gr  Unknown | | ___________gr  Unknown | | ____________gr  Unknown | ____________gr  Unknown | | | ___________gr  Unknown | | ____________gr  Unknown |
| High blood pressure | Yes  No | | Yes  No | | Yes  No | Yes  No | | | Yes  No | | Yes  No |
| Pre-eclampsia= high blood pressure + protein in the urine | Yes  No | | Yes  No | | Yes  No | Yes  No | | | Yes  No | | Yes  No |
| Gestational diabetes | Yes  No | | Yes  No | | Yes  No | Yes  No | | | Yes  No | | Yes  No |
| Placental abruption | Yes  No | | Yes  No | | Yes  No | Yes  No | | | Yes  No | | Yes  No |
| HELLP syndrome | Yes  No | | Yes  No | | Yes  No | Yes  No | | | Yes  No | | Yes  No |
| Child deceased in the womb | Yes  No | | Yes  No | | Yes  No | Yes  No | | | Yes  No | | Yes  No |
| During the pregnancy, I received medications for: | | | | | | | | | | | |
| High blood pressure | Yes  No | Yes  No | | Yes  No | | | Yes  No | Yes  No | | Yes  No | |
| Pre-eclampsia | Yes  No | Yes  No | | Yes  No | | | Yes  No | Yes  No | | Yes  No | |
| HELLP syndrome | Yes  No | Yes  No | | Yes  No | | | Yes  No | Yes  No | | Yes  No | |

**Supplement 3 - Missingness**

**S3T1. Information that could not be extracted from the EHR, and thus was assumed missing/not available.**

| Variable | Consent  N=2378 | Non-consent  N=1907 | Non-response  N=1445 |
| --- | --- | --- | --- |
| Age (%) | 0 | 0 | 0 |
| Sex (%) | 0 | 0 | 0 |
| Education (%) | 28 | 48 | 80 |
| Current Smoking (%) | 4 | 33 | 74 |
| Specialism (%) | 0 | 0 | 0 |
| Physical Activity per week (METminutes) (%) | 15 | 45 | 79 |
| Previous AMI, CABG, CHF, Stroke, ICH, TIA, PAD (IC, AAA, Carotid) (%) | 3 | 32 | 74 |
| - Previous AMI, CABG, arrest (%) | 3 | 32 | 74 |
| - Previous CHF (%) | 3 | 32 | 74 |
| - Previous stroke, ICH, TIA (%) | 3 | 32 | 74 |
| - Previous peripheral arterial disease (%) | 3 | 32 | 74 |
| Hypertension (%) | 3 | 32 | 74 |
| Kidney disease (%) | 3 | 32 | 74 |
| Proteinuria (%) | 3 | 32 | 74 |
| Diabetes (%) | 3 | 32 | 74 |
| Hyperlipidemia (%) | 18 | 29 | 81 |
| Body Mass Index (kg/m^2^) (%) | 6 | 12 | 52 |
| Heart rate (bpm) (%) | 5 | 9 | 53 |
| Systolic blood pressure (mmHg) (%) | 6 | 11 | 53 |
| Diastolic blood pressure (mmHg) (%) | 6 | 11 | 53 |
| Total Cholesterol (mmol/L) (%) | 17 | 27 | 80 |
| HDL-Cholesterol (mmol/L) (%) | 18 | 28 | 81 |
| Hb (mmol/L) (%) | 13 | 18 | 50 |
| Creatinine (µmol/L) (%) | 13 | 18 | 50 |
| eGFR (CKD epi) (%) | 13 | 18 | 50 |
| HbA1c (mmol/mol) (%) | 19 | 27 | 82 |
| 10-yr risk on CVD (%) | 29 | 59 | 98 |

*Notes:* N = number; % = percentage; OPD = outpatient department; MET = Metabolic Equivalent of Task; AMI = acute myocardial infarction; CABG = coronary artery bypass grafting; CHF = congestive heart failure; ICH = intracranial hemorrhage; TIA = transient ischemic attack; PAD = peripheral arterial disease; AAA = abdominal aortic aneurysm; bpm = beats per minute; SD = standard deviation; HDL = high-density lipoprotein; Hb = haemoglobin; eGFR = estimated glomerular filtration rate using the CKD-EPI formula; CKD = chronic kidney disease; HbA1c = glycated haemoglobin; yr = year.

**Supplement 4 – Patient inclusion flow-chart**

**
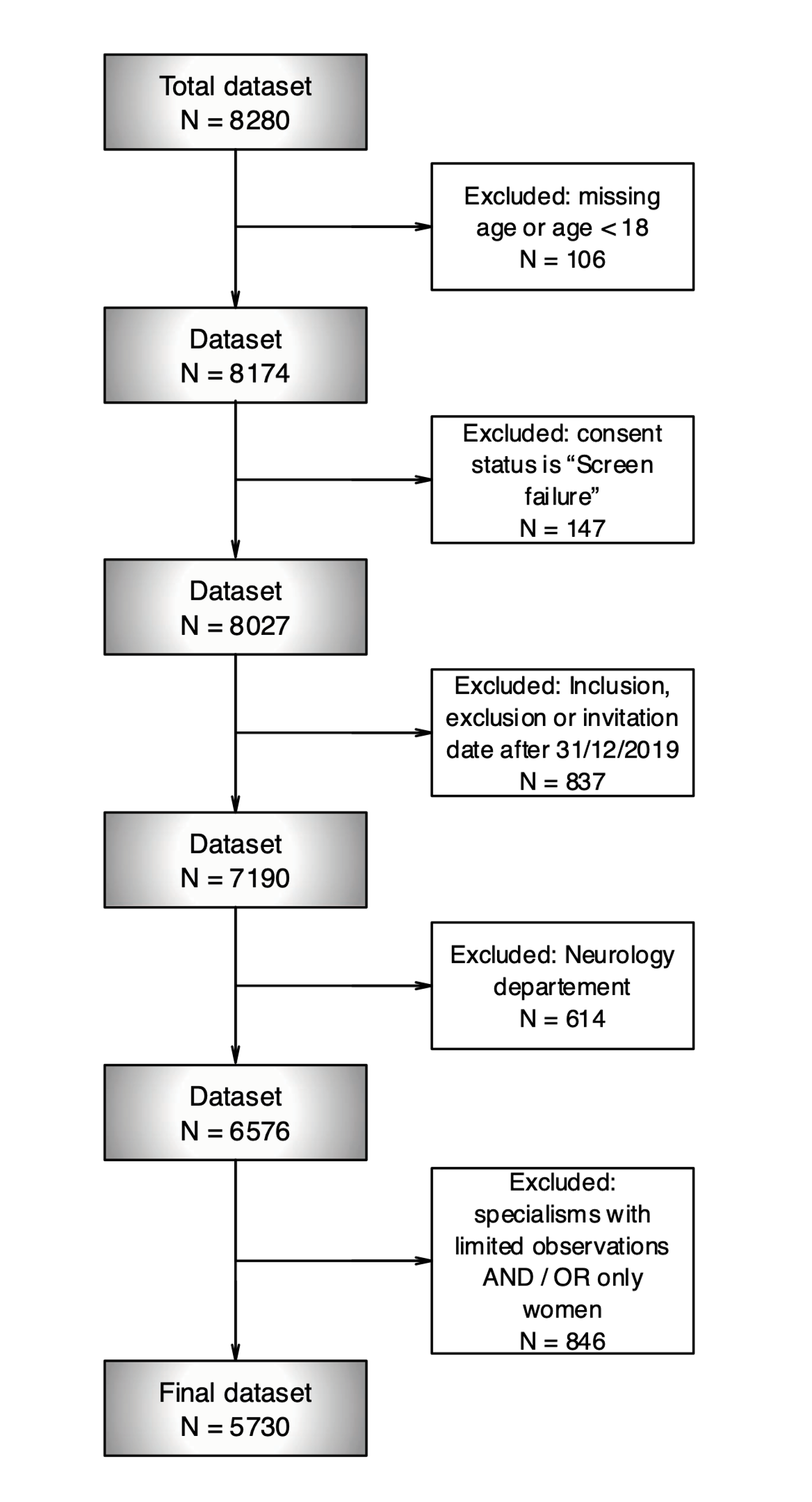
S4F1. Patient inclusion flow-chart**

**Supplement 5 – Sensitivity analysis to explore age distributions**

**S5F1. Age distribution across specialisms**


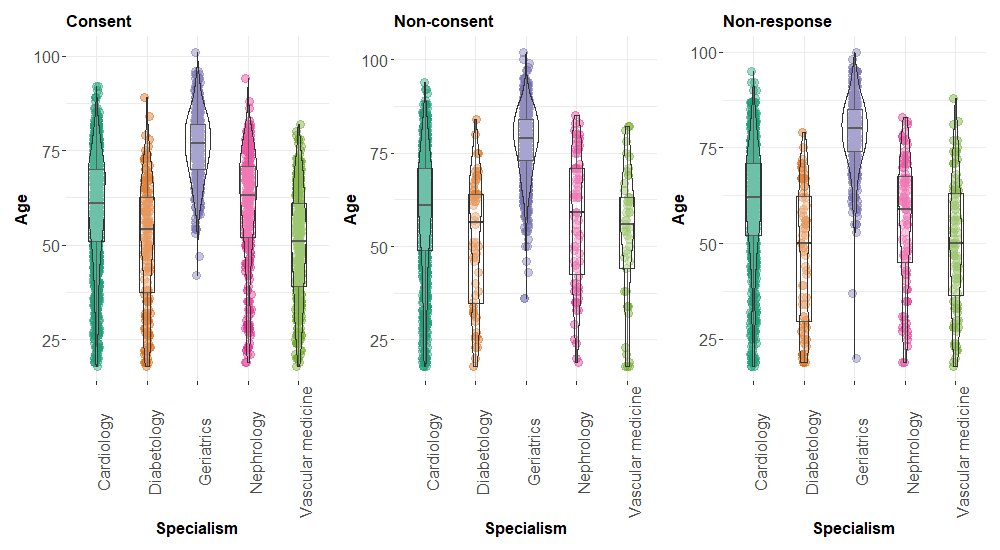


**S5T1. Number patients in each age group stratified by consent status in the total study population and the population excluding the geriatrics department.**

|  | Total population | | Population excluding Geriatrics | |
| --- | --- | --- | --- | --- |
|  | Consent | Non-consent | Consent | Non-consent |
| Age, N(%) |  |  |  |  |
| 18-29 yrs | 134 (65.0) | 72 (35.0) | 134 (65.0) | 72 (35.0) |
| 30-39 yrs | 151 (61.9) | 93 (38.1) | 151 (62.4) | 91 (37.6) |
| 40-49 yrs | 244 (68.5) | 112 (31.5) | 242 (68.8) | 110 (31.2) |
| 50-59 yrs | 453 (67.5) | 218 (32.5) | 440 (69.3) | 195 (30.7) |
| 60-69 yrs | 601 (61.4) | 378 (38.6) | 510 (67.0) | 251 (33.0) |
| 70-79 yrs | 539 (50.4) | 530 (49.6) | 347 (63.1) | 203 (36.9) |
| 80-89 yrs | 225 (33.8) | 440 (66.2) | 70 (49.3) | 72 (50.7) |
| 90 + yrs | 31 (32.6) | 64 (67.4) | 5 (55.6) | 4 (44.4) |
| Total | 2378 (55.5) | 1907 (44.5) | 1899 (65.6) | 998 (34.4) |

*Notes:* N = number, % = percentage; yrs = years.

**Supplement 6 – Determinants of non-consent**

**S6T1. Multivariable logistic regression analyses**

Table 1. Determinants of non-consent obtained by a multivariable logistic regression model, using a multiple imputed dataset.

|  |  | OR (95% CI) | P-value |
| --- | --- | --- | --- |
| Sex | men | 1.00 |  |
|  | women | 1.15 (0.99-1.33) | ns |
| Age | 70-79 yrs | 1.00 |  |
|  | 18-29 yrs | 1.41 (0.99-2.00) | ns |
|  | 30-39 yrs | 1.67 (1.20-2.32) | ** |
|  | 40-49 yrs | 1.13 (0.84-1.51) | ns |
|  | 50-59 yrs | 1.03 (0.82-1.31) | ns |
|  | 60-69 yrs | 0.96 (0.79-1.16) | ns |
|  | 80-89 yrs | 1.35 (1.08-1.67) | ** |
|  | 90 + yrs | 1.13 (0.71-1.79) | ns |
| Specialism | Cardiology | 1.00 |  |
|  | Diabetology | 0.43 (0.30-0.61) | *** |
|  | Geriatrics | 2.13 (1.79-2.55) | *** |
|  | Nephrology | 0.37 (0.27-0.49) | *** |
|  | Vascular medicine | 0.27 (0.20-0.38) | *** |
| Education level | low | 1.00 |  |
|  | high | 0.76 (0.60-0.97) | * |
| BMI (per kg/m2) |  | 0.97 (0.96-0.99) | *** |
| Physical activity (METmin / 1000) |  | 0.96 (0.94-0.98) | *** |
| Heart rate (bpm) | <60 | 1.00 |  |
|  | 60 >= & <= 100 | 1.20 (0.98-1.48) | ns |
|  | > 100 | 2.06 (1.42-2.98) | *** |
| HbA1c (mmol/mol) | < 42 | 1.00 |  |
|  | >= 42 & <= 47 | 1.05 (0.83-1.33) | ns |
|  | > 48 | 1.35 (1.08-1.70) | ** |
| Hb (mmol/L) |  | 0.82 (0.76-0.89) | *** |
| History with CVD: AMI, CABG, CHF, Stroke, ICH, TIA, PAD (IC, AAA, Carotid) | No | 1.00 |  |
|  | Yes | 1.43 (1.23-1.66) | *** |
| Proteinuria | No | 1.00 |  |
|  | Yes | 0.69 (0.55-0.87) | *** |

*Notes:* OR = odds ratio indicating the likelihood of having provided a non-consent as compared to a consent; % = percentage; CI = confidence interval; P-value = probability value assessed by multivariable analyses as described in the methods; yrs = years; BMI = Body Mass Index; METmin = metabolic equivalent of task-minutes; bpm = beats per minute; HbA1c = glycated haemoglobin; Hb = haemoglobin; CVD = cardiovascular disease; AMI = acute myocardial infarction; CABG = coronary artery bypass grafting; CHF = congestive heart failure; ICH = intracranial hemorrhage; TIA = transient ischemic attack; PAD = peripheral arterial disease; AAA = abdominal aortic aneurysm; ns P >.05; * P ≤ .05; ** P≤ .01; *** P ≤ .001.

**Supplement 7 – Determinants of non-response (exploratory analysis)**

**S7F1. Multivariable multinomial regression analyses.**

In this analysis missing values of the non-response group have been imputed in the same manner as the consent and the non-consent group described in the methods. Results from this multinomial regression analysis should be interpreted cautiously, due to the nature and the amount of missing data in the non-response group.

Figure 1. Multinomial regression analyses comparing the non-consent and the non-response group with the consent group.

**
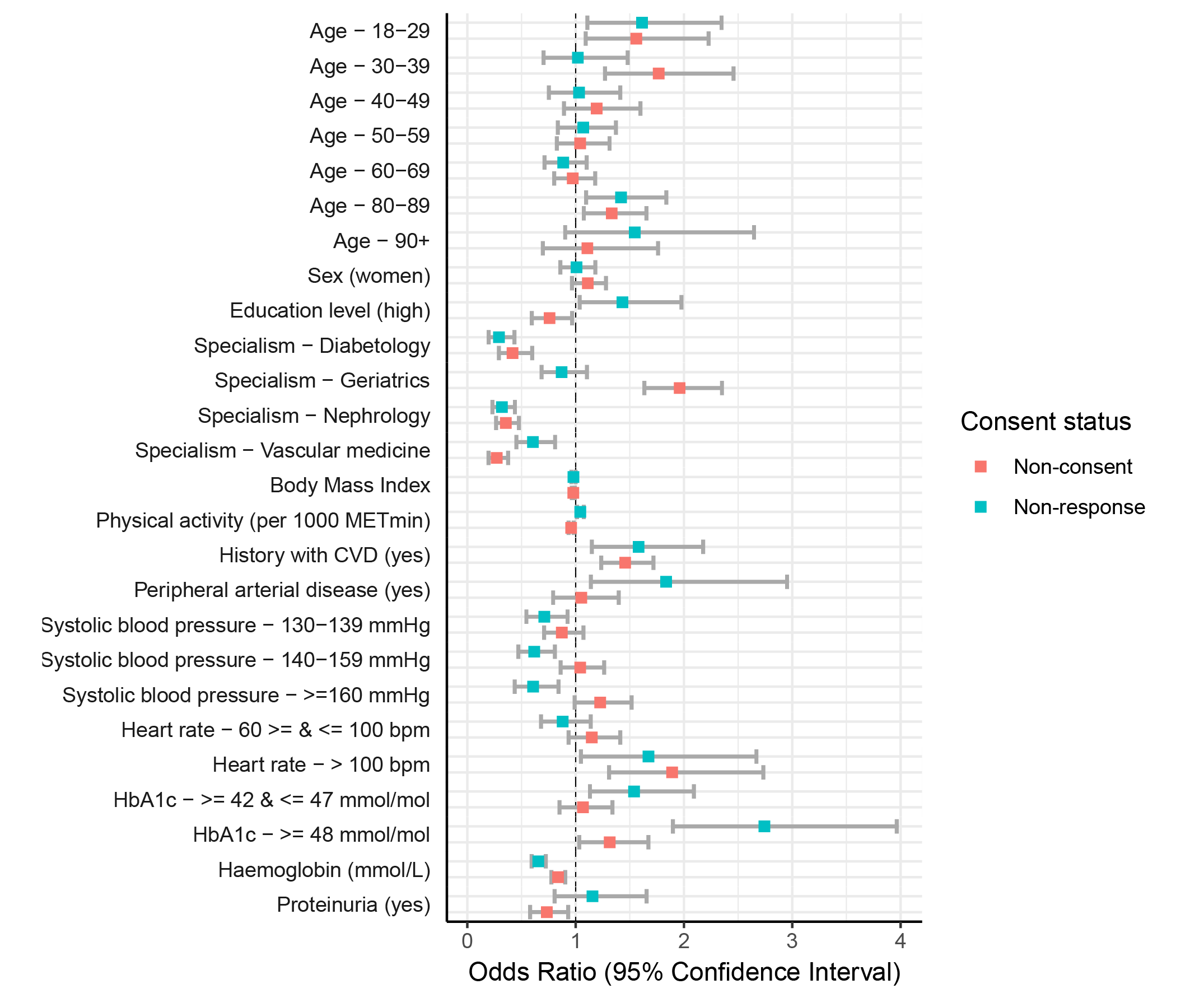
**

*Notes:* METmin = metabolic equivalent of task-minutes; CVD = cardiovascular diseases; bpm = beats per minute; HbA1c = glycated haemoglobin; OR = odds ratio indicating the likelihood of having provided a non-consent or non-response as compared to a consent; % = percentage.
